# Automated and code-free development of a risk calculator using ChatGPT-4 for predicting diabetic retinopathy and macular edema without retinal imaging

**DOI:** 10.1186/s40942-025-00638-9

**Published:** 2025-01-31

**Authors:** Eun Young Choi, Joon Yul Choi, Tae Keun Yoo

**Affiliations:** 1 Department of Ophthalmology, Institute of Vision Research, Gangnam Severance Hospital, Yonsei University College of Medicine, Seoul, South Korea; 2 Department of Biomedical Engineering, Yonsei University, Wonju, South Korea; 3 Department of Ophthalmology, Hangil Eye Hospital, 35 Bupyeong-Daero, Bupyeong-Gu, Incheon, 21388 South Korea

**Keywords:** Diabetic retinopathy, Diabetic macular edema, ChatGPT-4, No-code, Code-free prompt, Risk calculator

## Abstract

**Background:**

Diabetic retinopathy (DR) and macular edema (DME) are critical causes of vision loss in patients with diabetes. In many communities, access to ophthalmologists and retinal imaging equipment is limited, making screening for diabetic retinal complications difficult in primary health care centers. We investigated whether ChatGPT-4, an advanced large-language-model chatbot, can develop risk calculators for DR and DME using health check-up tabular data without the need for retinal imaging or coding experience.

**Methods:**

Data-driven prediction models were developed using medical history and laboratory blood test data from diabetic patients in the Korea National Health and Nutrition Examination Surveys (KNHANES). The dataset was divided into training (KNHANES 2017–2020) and validation (KNHANES 2021) datasets. ChatGPT-4 was used to build prediction formulas for DR and DME and developed a web-based risk calculator tool. Logistic regression analysis was performed by ChatGPT-4 to predict DR and DME, followed by the automatic generation of Hypertext Markup Language (HTML) code for the web-based tool. The performance of the models was evaluated using areas under the curves of receiver operating characteristic curve (ROC-AUCs).

**Results:**

ChatGPT-4 successfully developed a risk calculator for DR and DME, operational on a web browser without any coding experience. The validation set showed ROC-AUCs of 0.786 and 0.835 for predicting DR and DME, respectively. The performance of the ChatGPT-4 developed models was comparable to those created using various machine-learning tools.

**Conclusion:**

By utilizing ChatGPT-4 with code-free prompts, we overcame the technical barriers associated with using coding skills for developing prediction models, making it feasible to build a risk calculator for DR and DME prediction. Our approach offers an easily accessible tool for the risk prediction of DM and DME in diabetic patients during health check-ups, without the need for retinal imaging. Based on this automatically developed risk calculator using ChatGPT-4, health care workers will be able to effectively screen patients who require retinal examinations using only medical history and laboratory data. Future research should focus on validating this approach in diverse populations and exploring the integration of more comprehensive clinical data to enhance predictive performance.

**Graphical Abstract:**

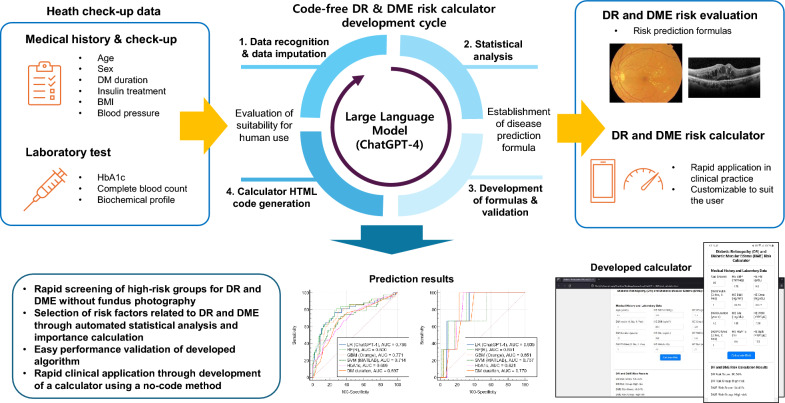

**Supplementary Information:**

The online version contains supplementary material available at 10.1186/s40942-025-00638-9.

## Introduction

Diabetes complications are a significant health problem worldwide for people with diabetes [1]. Diabetic retinopathy (DR) and diabetic macular edema (DME) are the most common vision-threatening conditions among diabetic patients. If DR and DME are diagnosed early, the progression of the disease can be effectively prevented by controlling blood sugar levels, administering anti-VEGF or corticosteroid injections, and performing laser treatments. However, if the disease progresses, ischemia in the retinal tissue worsens, and new blood vessels grow, leading to a proliferative stage. Early diagnosis is very important, as progression to proliferative diabetic retinopathy and ischemic maculopathy stages can result in permanent vision loss [2]. Therefore, it is recommended to undergo retinal examinations at least annually [3]. Follow-up care after diabetes diagnosis usually includes blood tests to monitor blood sugar control and related systemic conditions. However, due to the lack of access to ophthalmologists in most countries, screening for DR and DME, which requires immediate retinal examination, is often not performed properly.

To solve the problem of poor accessibility to eye clinics, studies have been conducted to identify risk groups for DR and DME that require retinal examinations based on medical history and laboratory blood test results [4, 5]. Since the pathogenesis of diabetic complications is multifactorial [6], a comprehensive analysis of healthcare data is necessary to predict DR and DME. For multivariate big data analysis, applying machine learning and deep learning models is more effective than using traditional statistical formulas. However, developing a model suitable for addressing the specific tasks of each clinical department requires complex coding skills, which poses a challenge for health care workers. Additionally, there are significant difficulties in directly applying the developed models to clinical settings [7]. Most machine learning algorithms require downloading software with the trained model or accessing it through a server computer, which makes it difficult for clinicians to use [8]. Differences in medical history profiles and laboratory tests between clinics, rising health care costs, and clinic overcrowding may also cause limited use of AI in clinical practice. The AI algorithms can only be used clinically if a user interface is provided in the form of a calculator that incorporates the formulas.

**Fig. 1:**
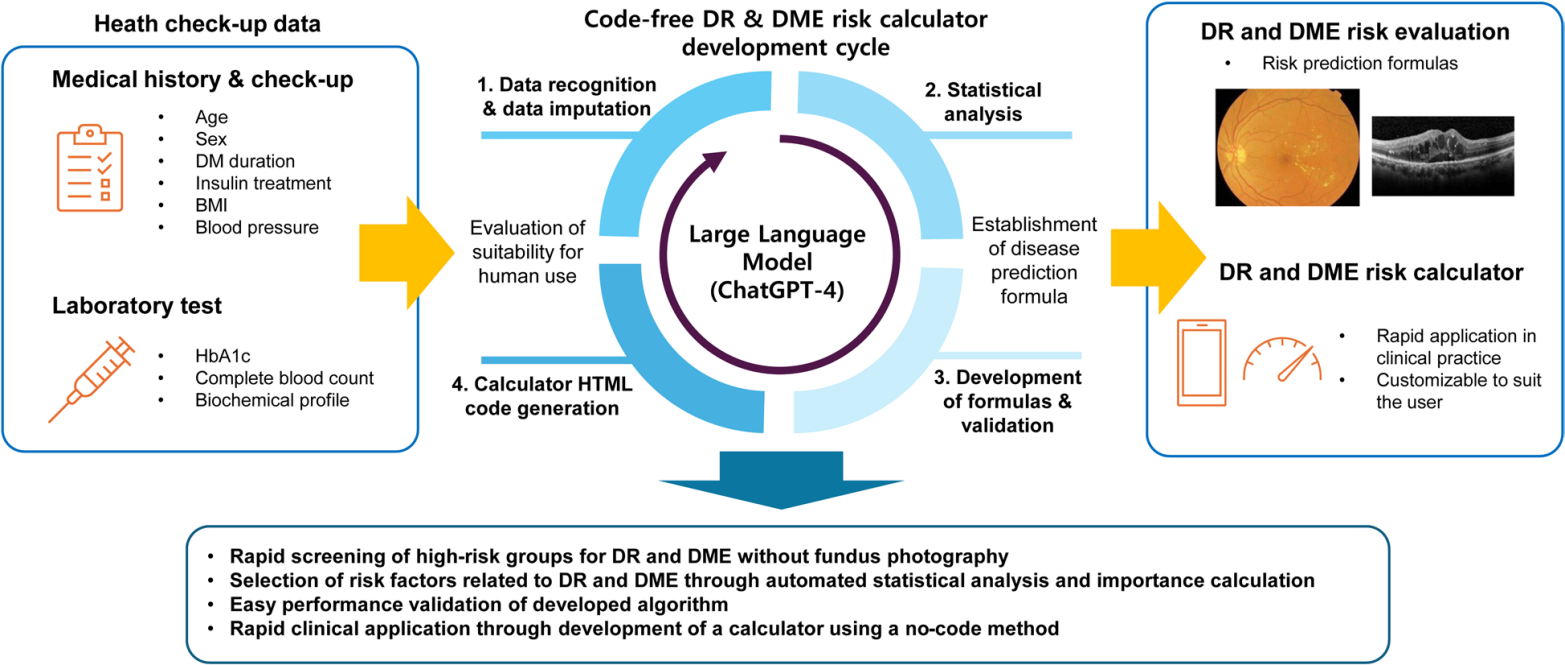
Schematic diagram of this study

Recent developments in large language models (LLMs) have enabled data analysis and software development without coding [9]. ChatGPT-4, an advanced multimodal LLM by OpenAI, is capable of understanding and generating human-like text, making it a powerful tool for tasks such as natural language processing, data analysis, and even software development without the need for traditional coding skills. Although numerous functions of LLM are being introduced in ophthalmology [10], research on their application in data analysis within the field remains limited. In this study, using ChatGPT-4, we automatically developed a risk calculator to screen for DR and DME risk groups in diabetic patients using data from the Korea National Health and Nutrition Examination Surveys (KNHANES). Through this study, we aimed to present a ChatGPT-4 based framework that can be easily applied in various medical fields to calculate the risk of major diseases without the need for a coding process (Fig. 1).

## Methods

### Dataset

To construct a risk prediction model for DR and DME, we utilized data from diabetic patients collected through the Korea National Health and Nutrition Examination Survey (KNHANES). Conducted nationwide by the Korea Disease Control and Prevention Agency (KDCA), the KNHANES is a cross-sectional survey. The study protocol received approval from the KDCA Institutional Review Board, and informed consent was secured from all participants prior to their involvement. The dataset is publicly accessible for research purposes at https://knhanes.kdca.go.kr/knhanes/eng/index.do. This research complied with the principles outlined in the Declaration of Helsinki. Participants were selected using stratified random sampling to ensure representation based on factors such as sex, age, and residential area [11]. The dataset includes health records derived from interviews covering medical history, health behaviors, and nutrition surveys, alongside sociodemographic data, laboratory test results, and ophthalmologic examination outcomes [12]. Laboratory assessments comprised standard blood tests and biochemical profiles, conducted after an overnight fast.

The input data for model development included age, body mass index (BMI), waist circumference, household income level, smoking status, systolic blood pressure (SBP), diastolic blood pressure (DBP), duration of diabetes, use of oral or injectable antidiabetic drugs, and laboratory test results. The laboratory tests included white blood cell (WBC) count, hemoglobin, platelet counts, fasting glucose, total cholesterol, triglyceride (TG), aspartate aminotransferase (AST), alanine aminotransferase (ALT), creatinine, and uric acid.

**Fig. 2:**
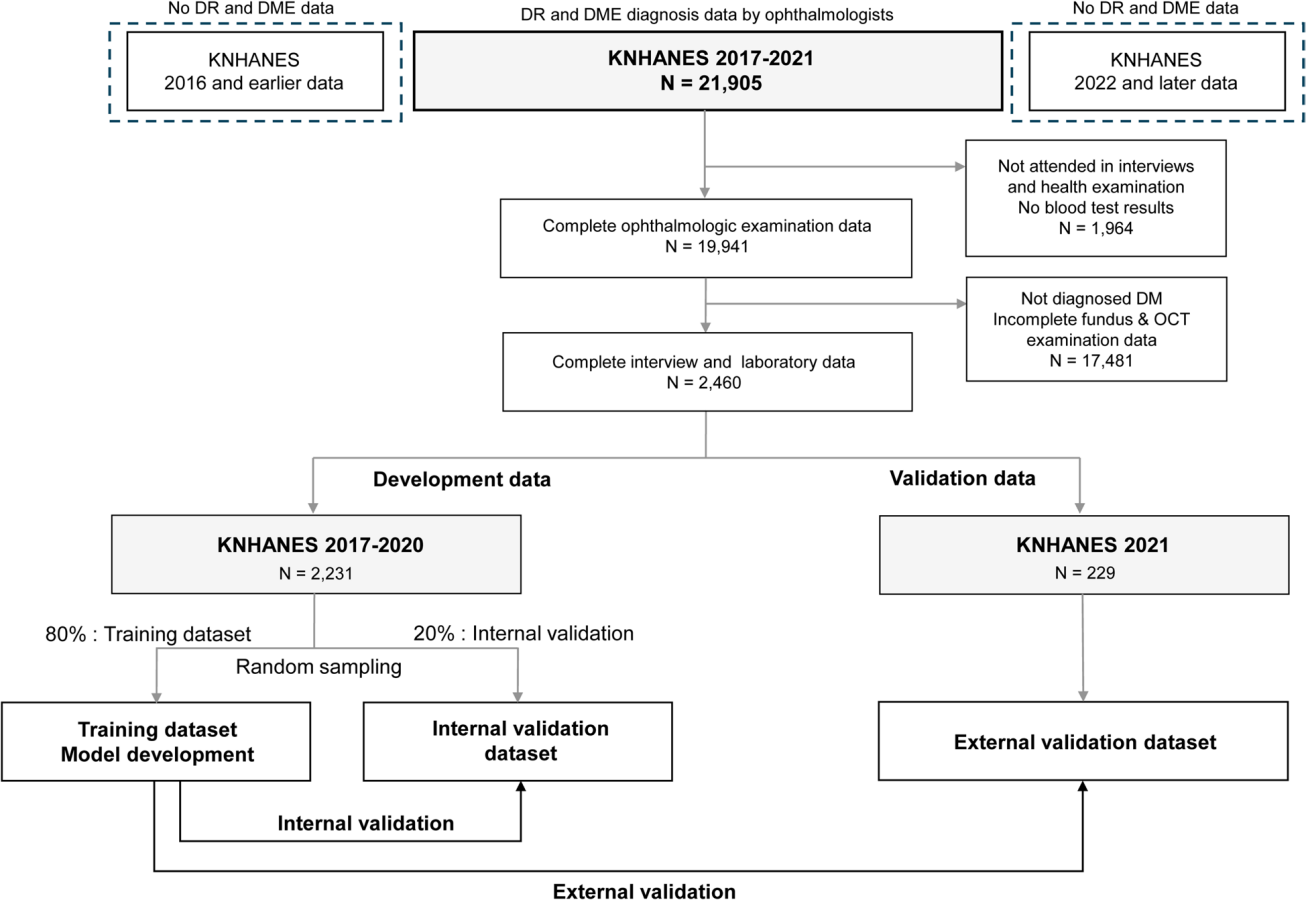
Inclusion and exclusion of data in this study

The data workflow is illustrated in Fig. 2. We selected the KNHANES data of the 2017–2021 period because retinal disease grading was conducted based on retinal imaging within this 5-year period. Diabetes was defined by a fasting blood glucose ≥ 126 mg/dL, glycated hemoglobin (HbA1c) ≥ 6.5%, a diagnostic history of diabetes, or using any oral or injectable antidiabetic drugs. We established a study design to develop and validate prediction models in chronological order, with data split by calendar time. The DR and DME prediction models were developed using KNHANES data from 2017 to 2020 as the development dataset. Because the KNHANES randomly resamples participants yearly, the performance of the developed prediction models was evaluated using an independent dataset from KNHANES 2021. Accordingly, we used KNHANES 2021 data for external validation. Within the development sets, 80% of the data were randomly selected and used as the training dataset, while the remaining 20% were used as the internal validation dataset. Model training and validation were performed without normalizing the input variables.

### Definition of diabetic retinopathy and macular edema

In the KNHANES, macular optical coherence tomography (OCT; Cirrus HD-OCT 500, Carl Zeiss Meditec, Jena, Germany) and non-mydriatic fundus photography (VISUCAM, Carl Zeiss Meditec) were utilized. DR and DME were evaluated by retinal specialists certified by the Korean Retina Society. Each fundus photograph and OCT image was independently assessed twice by an independent grader. In cases where there was disagreement in the initial diagnosis, a reading committee from the Korean Retina Society reviewed the images to establish a final consensus. The procedures and criteria used to define DR and DME in the KNHANES have been elaborated in prior research [13, 14]. Clinically significant macular edema (CSME) was defined according to the following criteria, which include retinal thickening (≥ 300 microns) within 500 microns of the macular center, hard exudates within 500 microns of the macular center if associated with adjacent retinal thickening, or retinal thickening measuring one disc area or larger, with any part located within one disc diameter of the macular center [13, 15]. Additionally, eyes that lacked typical CSME features but showed foveal thickening exceeding 300 microns, as documented by OCT within the ETDRS grid, were also classified as having cystoid macular edema. The Epidemiologic Survey Committee of the Korean Ophthalmologic Society (KOS) ensured the quality of eye examinations. To maintain consistency, members of the National Epidemiologic Survey Committee of the KOS regularly provided training to participating ophthalmologists and residents. The Korea Disease Control and Prevention Agency (KDCA) validated both the data collection protocols and overall data quality.

### Risk calculator development using ChatGPT-4

Table 1 provides a comprehensive overview of the prompts used for performing logistic regression and constructing the DR and DME risk calculator. To begin, the training dataset was uploaded to ChatGPT-4 by dragging the file into the chat window. Logistic regression analysis was then conducted to predict DR, with input variables specified by listing the column names from the CSV file and clearly identifying the diabetic retinopathy variable as the target [16]. Feature selection was carried out concurrently using a backward elimination approach during the regression process.

**Table 1:** Prompts used to develop a risk calculator to predict diabetic retinopathy and macular edema

Tasks	Prompts
1. Load the training dataset	(After dragging the training dataset into the dialog window) This data file is designed to predict diabetic retinopathy and diabetic macular edema using patients’ medical history and laboratory data
2. Develop DR prediction formula	Develop a formula to predict the occurrence of diabetic retinopathy using the following items The variable targeted for prediction is E_DR (whether diabetic retinopathy occurs) Please use logistic regression analysis using backward elimination feature selection method Enter the following variables as input: Sex, age, ho_incm, Dyslipidemia, DM_insulin, DM_duration, DM_po_med, Smoking, HE_sbp, HE_dbp, HE_wc, HE_BMI, HE_glu, HE_HbA1c, HE_chol, HE_TG, HE_ast, HE_alt, HE_HB, HE_BUN, HE_crea, HE_WBC, HE_Bplt, HE_Uacid
	Please show the final prediction formula to predict E_DR
	Draw the ROC curve of the E_DR prediction formula using the formula developed above. Show the optimal cutoff value
3. Develop DME prediction formula	Develop a formula to predict the occurrence of diabetic macular edema using the following items The variable targeted for prediction is E_DME (whether diabetic macular edema occurs) Please use logistic regression analysis using backward elimination feature selection method Enter the following variables as input: Sex, age, ho_incm, Dyslipidemia, DM_insulin, DM_duration, DM_po_med, Smoking, HE_sbp, HE_dbp, HE_wc, HE_BMI, HE_glu, HE_HbA1c, HE_chol, HE_TG, HE_ast, HE_alt, HE_HB, HE_BUN, HE_crea, HE_WBC, HE_Bplt, HE_Uacid
	Please show the final prediction formula to predict E_DR
	Draw the ROC curve of the E_DME prediction formula using the formula developed above. Show the optimal cutoff value
4. Organize formulas and input data	Please describe the formulas to predict E_DR (diabetic retinopathy) and E_DME (diabetic macular edema), which were developed above. Show the cutoff values, mentioned above, of each formula to identify the high-risk groups
	What are the variables needed to calculate these formulas above?
5. Create HTML codes to build a risk calculator	Create a calculator that calculates the percentage risk scores of E_DR and E_DME using above formulas Build the codes for the risk calculator written in html, css, and javascript in one html file Design: The text boxes must have rounded edges Make this calculator look professional by creating a frame around it Separate the title, input window and output window frames Insert the title of the calculator in the title frame above the input window frame. The title is "Diabetic retinopathy (DR) and diabetic macular edema (DME) risk calculator" Add the subtitle of the input window inside the frame: “Medical history and laboratory data” Add the subtitle of the output window inside the frame: “DR and DME risk results” The input frame should be above, and the output frame should be below. The title, input, and output frames must be aligned in order from the top Input items: Above-mentioned variables to calculate the formulas Please show the units of the variables Output items: DR risk score (%) Whether the calculation result corresponds to the DR high-risk or low-risk group DME risk score (%) Whether the calculation result corresponds to the DME high-risk or low-risk group
	Set the size of the calculator to 800 by 800 pixels. Input items are in three columns. Please keep the inputs and calculation information as are
6. Validation of the developed DR and DME formulas	(After dragging the test dataset into the dialog window) This is the dataset for the external validation. Please draw ROC curves of the developed formulas above to predict DR and DME. Calculate the sensitivity and specificity at the optimal cutoff using the Youden's index

*DME* diabetic macular edema, *DR* diabetic retinopathy, *ho_incm* household income, *HE_sbp* systolic blood pressure, *HE_dbp* diastolic blood pressure, *HE_wc* waist circumference, *HE_BMI* body mass index, *HE_glu* fasting glucose level, *HE_chol* total cholesterol, *HE_TG* Triglyceride, *HE_ast* aspartate aminotransferase, *HE_alt* alanine aminotransferase, *HE_HB* hemoglobin, *HE_BUN* blood urea nitrogen, *HE_crea* creatinine, *HE_WBC* white blood cell count, *HE_Bplt* platelet count, *HE_Uacid* uric acid

Subsequently, we prompted ChatGPT-4 to explain the derived formulas within the chat interface, and the prediction performance was assessed through a receiver operating characteristic (ROC) curve. Validation datasets were similarly uploaded by dragging files into the chat window. Since ChatGPT-4 automatically identified the column names, no additional manipulation of variables or files was necessary.

After establishing the prediction formulas for DR and DME, cutoff values for each prediction were verified, and the input variables were organized to facilitate the development of a calculator. Using these specifications, ChatGPT-4 was instructed to create DR and DME risk calculators in Hypertext Markup Language (HTML). The HTML code enabled the calculators to function as webpages, executable through any standard web browser. To improve the user interface, additional prompts were used to define specific design features of the calculator. The final code was saved as a single HTML file and successfully run in a web browser.

### Comparison analysis

The performance of DR and DME prediction models developed in this study was compared with that of other machine learning algorithms using the same training set. The evaluation was conducted on both internal and external validation datasets. We adopted machine learning algorithms, including random forest (RF) with R version 4.2.1 (The Comprehensive R Archive Network; http://cran.r-project.org) [17], gradient boosting machine (GBM) using Orange Data Mining version 3.36.2 (Bioinformatics Laboratory, University of Ljubljana, Ljubljana, Slovenia) [18], and support vector machine (SVM) with a radial basis function kernel using MATLAB 2022a (The MathWorks Inc., Natick, MA, USA) [19, 20]. These algorithms were selected based on their empirically demonstrated excellent performance in disease prediction. A grid search was conducted to evaluate the range of tunable parameter values with internal validation to obtain the best hyperparameters for each algorithm. In addition, HbA1c levels and disease duration, which are known to be important factors in the severity of diabetes, were compared with the predicted results as independent indicators [21].

### Statistical analysis

The results of the prediction models were evaluated using the area under the ROC curve (AUCs). Data were compared using the chi-square test for categorical variables and Student’s t-test for continuous variables. The maximum Youden’s index was used to determine the cutoff value. All tests were two-sided, with a significance level of P value < 0.05. All ROC curve analyses were performed using MedCalc Version 22.021 (Mariakerke, Belgium).

## Results

### Demographics

This study included 2,231 patients with diabetes in the development dataset and 229 patients in the external validation dataset. Of the development dataset, 1,785 patients were used for training, and 446 were used for the internal validation. The characteristics and laboratory data of patients with diabetes in this study are summarized in Table 2. Patients with DR had a longer duration of diabetes and a higher frequency of diabetes treatment (both oral medication and insulin treatment); higher SBP, HbA1c levels, and fasting glucose levels; and lower total cholesterol, AST, and hemoglobin levels compared to those without DR. Patients with DME were older; had a longer duration of diabetes and a higher frequency of diabetes treatment; higher SBP, HbA1c levels, and fasting glucose levels; and lower DBP, total cholesterol, AST, ALT, and hemoglobin levels compared to those without DME.

**Table 2:** Demographic and laboratory data of the diabetic patients included in this study

Variable	DR			DME		
	DR group (N = 503)	No DR group (N = 1957)	P-value	DME group (N = 37)	No DME group (N = 2423)	P-value
Age (years)	62.65 (10.32)	62.80 (11.18)	0.772	67.32 (10.63)	62.70 (11.00)	0.012
Sex (female)	236 (46.9%)	970 (49.5%)	0.312	17 (45.9%)	1189 (49.0%)	0.832
BMI (kg/m^2^)	25.03 (3.50)	25.61 (3.58)	0.001	24.80 (3.46)	25.50 (3.58)	0.153
Waist circumference (cm)	89.76 (9.41)	88.88 (9.11)	0.089	89.52 (9.35)	88.95 (9.29)	0.566
Current smoker	111 (22.0%)	363 (18.5%)	0.085	6 (16.22%)	468 (19.3%)	0.791
House income (lowest quartile)	150 (29.9%)	578 (29.6%)	0.913	15 (40.5%)	713 (29.5%)	0.150
DM history						
DM duration (years)	10.94 (10.02)	5.87 (7.72)	< 0.001	17.00 (11.01)	6.75 (8.36)	< 0.001
DM oral medication	397 (78.9%)	1174 (59.9%)	< 0.001	35 (94.5%)	1536 (63.3%)	< 0.001
DM insulin treatment	63 (12.52%)	43 (2.20%)	< 0.001	8 (21.62%)	98 (4.04%)	< 0.001
Blood pressure						
SBP (mmHg)	127.46 (16.50)	125.46 (15.81)	0.014	131.61 (16.68)	125.78 (15.95)	0.041
DBP (mmHg)	74.71 (10.53)	75.24 (10.18)	0.307	71.78 (9.97)	75.19 (10.25)	0.046
Laboratory tests						
HbA1c (%)	7.73 (1.57)	7.02 (1.13)	< 0.001	8.15 (1.63)	7.15 (1.25)	< 0.001
Fasting Glucose (mg/dL)	151.36 (49.43)	133.47 (33.32)	< 0.001	156.41 (57.56)	136.84 (37.43)	0.046
Total cholesterol (mg/dL)	170.81 (42.66)	176.26 (42.30)	0.010	163.32 (33.21)	175.33 (42.53)	0.036
Triglyceride (mg/dL)	162.02 (133.10)	160.74 (120.96)	0.845	152.81 (83.80)	161.13 (124.03)	0.556
AST (IU/L)	25.94 (18.48)	27.86 (14.50)	0.030	21.78 (6.09)	27.56 (15.50)	< 0.001
ALT (IU/L)	26.47 (27.17)	28.91 (21.44)	0.062	19.65 (10.20)	28.55 (22.85)	< 0.001
BUN (mg/dL)	17.21 (5.95)	16.74 (5.34)	0.106	20.70 (7.70)	16.78 (5.41)	0.003
Creatinine (mg/dL)	0.88 (0.45)	0.84 (0.24)	0.061	1.09 (0.73)	0.84 (0.28)	0.047
Uric acid (mg/dL)	4.95 (1.45)	5.07 (1.38)	0.110	5.26 (1.40)	5.04 (1.39)	0.345
WBC (10^3^ cells/μL)	6.72 (1.83)	6.61 (1.78)	0.209	7.16 (1.81)	6.62 (1.79)	0.079
Platelet (10^9^/L)	245.63 (61.94)	248.68 (66.55)	0.332	237.70 (63.18)	248.21 (65.67)	0.322
Hemoglobin (mg/dL)	13.86 (1.66)	14.05 (1.60)	0.023	13.28 (1.61)	14.02 (1.61)	0.007

*AST* aspartate aminotransferase, *ALT* alanine aminotransferase, *BMI* body mass index, *BUN* blood urea nitrogen, *DBP* diastolic blood pressure, *DM* diabetes mellitus, *DME* diabetic macular edema, *DR* diabetic retinopathy, *SBP* systolic blood pressure, *WBC* white blood cell count

### Development of a risk calculator

**Fig. 3:**
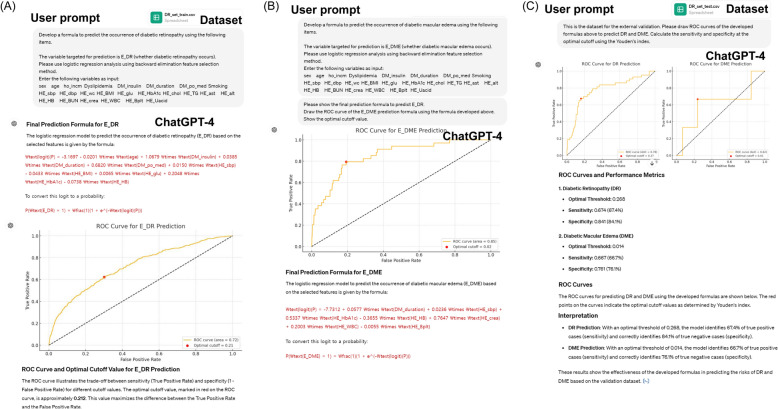
Screenshot of examples of regression analysis performed by ChatGPT-4. **A** Loading a dataset and development of a formula. **B** Development of new formulas based on context without additional data loading. **C** Data loading and evaluation for external validation

During the development process to build prediction models using ChatGPT-4, the researchers used formal English and did not perform coding or mathematical calculations themselves. ChatGPT-4 successfully recognized the training data in a CSV file and understood the meaning of the column names. Following the prompts provided, ChatGPT-4 performed logistic regression with feature selection to build formulas for DR and DME predictions (Fig. 3). The results of the logistic regression analysis with feature selection performed using ChatGPT-4, along with the calculated odds ratios, are presented in Table 3. To predict DR, the final model included the variables: age, BMI, duration of diabetes, oral medication, insulin treatment, SBP, HbA1c, fasting glucose, and hemoglobin levels. For predicting DME, the formulas included the variables: diabetes duration, SBP, HbA1c, creatinine, WBC count, platelet count, and hemoglobin level. The exact process was followed for predicting DME within the same dialog window without entering a new dataset.

**Table 3:** Logistic regression with feature selection performed by ChatGPT-4 for diabetic retinopathy using the training dataset

	Diabetic retinopathy			Diabetic macular edema		
	Coefficient	OR (95% CI)	P-value	Coefficient	OR (95% CI)	P-value
Age (years, per 1 unit increase)	−0.020	0.98 (0.97–0.99)	0.002	–	–	–
BMI (kg/m^2^, per 1 unit increase)	−0.043	0.96 (0.93–0.99)	0.011	–	–	–
DM duration (years, per 1 unit increase)	0.038	1.04 (1.02–1.06)	< 0.001	0.058	1.06 (1.04–1.10)	< 0.001
DM oral medication	0.682	1.97 (1.37–2.44)	< 0.001	–	–	–
DM insulin treatment	1.068	2.91 (1.93–4.75)	< 0.001	–	–	–
SBP (mmHg, per 1 unit increase)	0.015	1.02 (1.00–1.03)	0.001	0.024	1.03 (1.01–1.05)	0.014
HbA1c (%, per 1 unit increase)	0.205	1.23 (1.11–1.39)	< 0.001	0.534	1.70 (1.27–2.00)	< 0.001
Fasting glucose (mg/dL, per 1 unit increase)	0.007	1.01 (1.00–1.01)	0.002	–	–	–
Creatinine (mg/dL, per 1 unit increase)	–	–	–	0.765	2.15 (1.38–3.03)	0.021
WBC (cells/μL, per 1 unit increase)	–	–	–	0.200	1.22 (1.02–1.51)	0.033
Platelet (10^9^/L, per 1 unit increase)	–	–	–	−0.006	0.99 (0.98–0.99)	0.025
Hemoglobin (mg/dL, per 1 unit increase)	−0.074	0.93 (0.86–0.99)	0.034	−0.366	0.79 (0.57–0.92)	0.007
Constant	−3.167	–	0.002	−7.731	–	< 0.001

^*^Multivariate logistic regression analysis using all variables

^†^Multivariable logistic regression with stepwise backward selection model

**Fig. 4:**
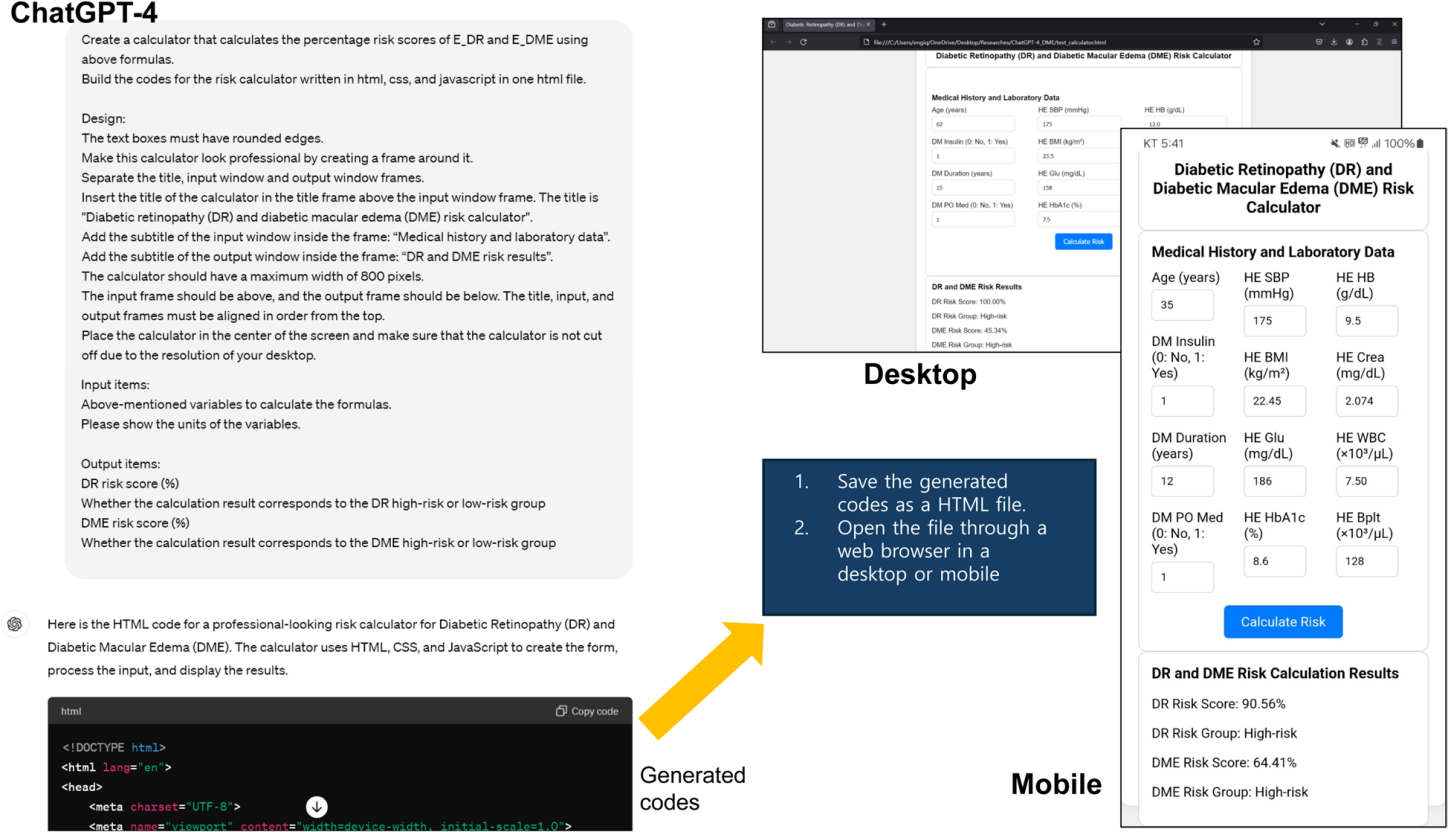
Risk calculator development process. The HTML code generated by ChatGPT-4 was opened in a web browser to run the developed calculator. This calculator is available at https://taekeuntoo.github.io/DR_DME_risk_calc/. *HE_sbp* systolic blood pressure, *HE_BMI* body mass index, *HE_glu* fasting glucose level, *HE_HB* hemoglobin, *HE_crea* creatinine, *HE_WBC* white blood cell count, *HE_Bplt* platelet count

After establishing all the formulas to predict DR and DME, we immediately instructed ChatGPT-4 to build a calculator using HTML (Fig. 4). ChatGPT-4 automatically generated HTML code that can be executed on a web browser to create a risk calculator. As shown in Table 1, the code was generated based on the context of previous conversations, and the input, output, and mathematical formulas were not entered separately into the prompt. The codes generated by ChatGPT-4 are shown in the Supplementary Materials. Using Calculator, users can run the saved HTML code file on a web browser. The calculator worked well without errors in both the desktop and mobile environments. This calculator is available at https://taekeuntoo.github.io/DR_DME_risk_calc/.

### Comparison of prediction models

**Fig. 5:**
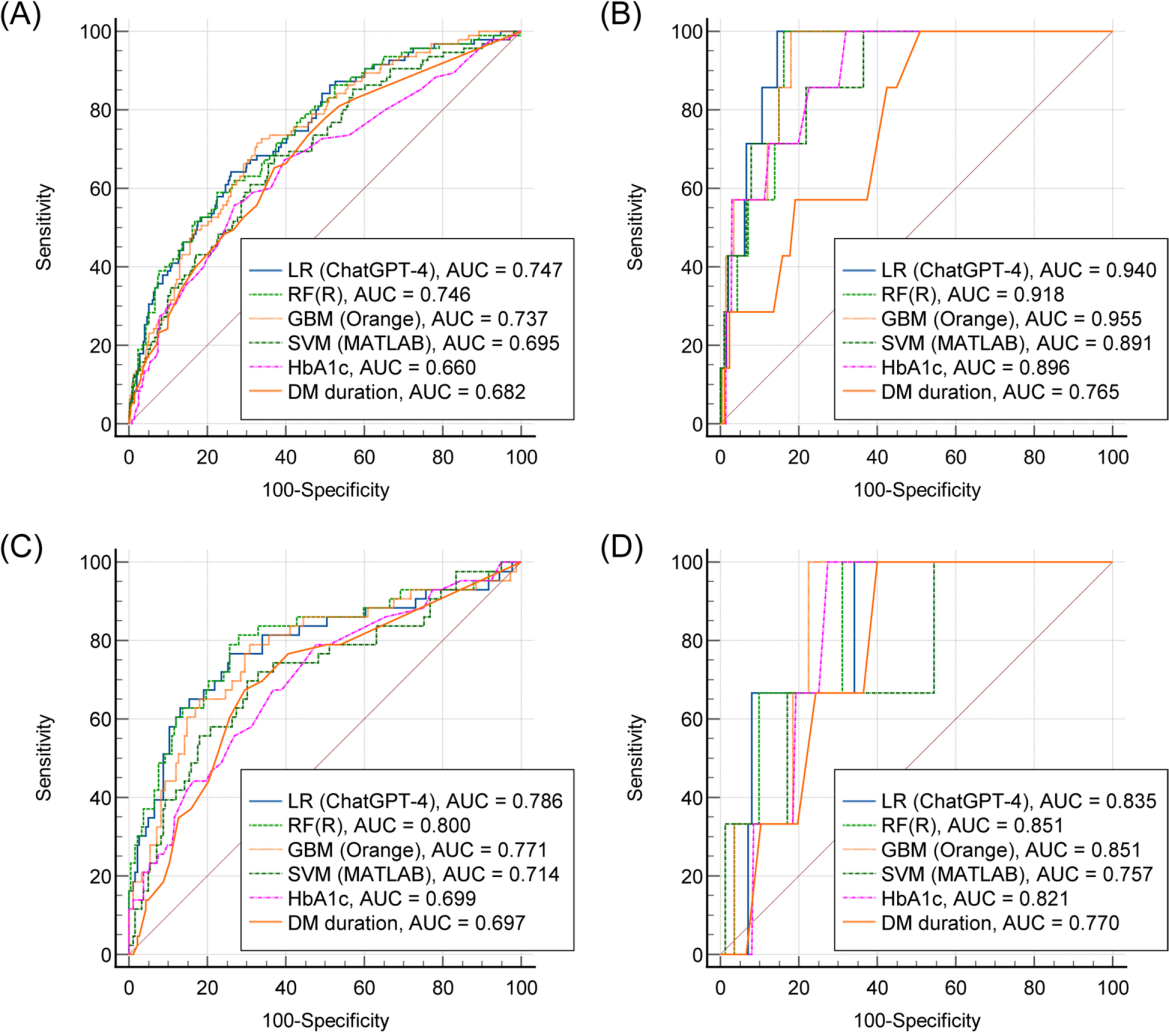
ROC curves for diabetic retinopathy (DR) and diabetic macular edema (DME) prediction. **A** DR prediction in the internal validation set. **B** DME prediction in the internal validation set. **C** DR prediction in the external validation set. **D** DR prediction in the external validation set

We evaluated the performance of the formulas established by ChatGPT-4 by comparing them with machine learning algorithms developed using various tools. Figure 5 shows the ROC curves for internal and external validations. In the internal validation set, the developed formula exhibited ROC AUCs of 0.747 and 0.940 for predicting DR and DME, respectively. In the external validation set, the formula showed an ROC-AUCs of 0.786 and 0.835 for predicting DR and DME, respectively. The fine-tuned RF from R exhibited the best performance in predicting DR (AUC = 0.800) and DME (AUC = 0.851) in the external validation.

Table 4 shows the detailed performance metrics used to predict DR. In the prediction of DR, the logistic regression formula from ChatGPT-4 showed no significant difference compared to RF and GBM in internal and external validations. Logistic regression outperformed SVM in the internal validation and diabetes indicators (HbA1c and diabetes duration) in internal and external validations. Table 5 lists the performance metrics used to predict the DME. In terms of DME, the logistic regression formula from ChatGPT-4 showed no significant difference compared to the other machine learning algorithms in both internal and external validations. It showed high predictive performance compared to diabetes indicators but did not show significance due to the small sample size. Diabetes duration and HbA1c levels were identified as important factors in feature selection for both RF (Supplementary Material 2) and GBM (Supplementary Material 3), and there was no significant difference from the feature selection results constructed by ChatGPT-4.

**Table 4:** Prediction performance of developed algorithms and diabetes indicators for diabetic retinopathy

	ROC-AUC [95% CI]	Accuracy (%), [95% CI]	Sensitivity (%), [95% CI]	Specificity (%), [95% CI]	P-value*
Internal validation					
LR (ChatGPT-4)	0.747 [0.703, 0.786]	72.0 [67.6, 76.1)	64.2 [53.7, 73.8)	74.1 [69.2, 78.7]	Reference
RF (R)	0.746 [0.703, 0.786]	73.3 [68.9, 77.4]	58.9 [48.3, 68.9]	77.3 [72.5, 81.6]	0.995
GBM (Orange)	0.737 [0.694, 0.778]	68.4 [63.8, 72.7]	71.6 [61.4, 80.3]	67.5 [62.3, 72.4]	0.169
SVM (MATLAB)	0.695 [0.650, 0.738]	64.1 [59.4, 68.5]	68.4 [58.1, 77.6]	62.9 [57.6, 68.0]	0.008
HbA1c	0.660 [0.616, 0.706]	69.5 [65.0, 73.8]	55.8 [45.2, 65.9]	73.2 [68.2, 77.8]	0.002
DM duration	0.682 [0.637, 0.725]	63.4 [58.7, 67.9]	65.2 [54.8, 74.7]	62.9 [57.6, 68.0]	0.004
External validation					
LR (ChatGPT-4)	0.786 [0.726, 0.837]	74.4 [68.3, 79.9]	77.2 [62.1, 88.5]]	73.7 [66.7, 79.9]	Reference
RF (R)	0.800 [0.742, 0.851]	74.0 [67.8, 79.6]	81.8 [67.3, 91.8]	72.1 [65.0, 78.5]	0.194
GBM (Orange)	0.771 [0.710, 0.824]	70.9 [64.6, 76.7]	79.5 [64.7, 90.2]	68.8 [61.6, 75.5]	0.168
SVM (MATLAB)	0.714 [0.650, 0.772]	69.6 [63.1, 75.5]	70.4 [54.8, 83.2]	69.4 [62.1, 75.9]	0.051
HbA1c	0.699 [0.635, 0.758]	57.7 [51.0, 64.2]	79.5 [64.7, 90.2]	52.5 [44.9, 59.9]	0.034
DM duration	0.697 [0.632, 0.756]	62.9 [56.3, 69.3]	77.2 [62.1, 88.5]	59.5 [52.1, 66.7]	0.029

*CI* confidence interval, *DM* diabetes mellitus, *GBM* gradient boosting machine, *LR* logistic regression, *ROC-AUC* area under the receiver operating characteristic curve, *SVM* support vector machine

^*^Differences in ROC-AUC values compared to the logistic regression performed using ChatGPT-4

**Table 5:** Prediction performance of developed algorithms and diabetes indicators for diabetic macular edema

	ROC-AUC [95% CI]	Accuracy (%), [95% CI]	Sensitivity (%), [95% CI]	Specificity (%), [95% CI]	P-value*
Internal validation					
LR (ChatGPT-4)	0.940 [0.914, 0.960]	85.6 [82.0, 88.7]	100.0 [59.0, 100.0]	85.4 [81.7, 88.5]	Reference
RF (R)	0.918 [0.888, 0.942]	84.0 [80.3, 87.3]	100.0 [59.0, 100.0]	83.8 [79.9, 87.1]	0.261
GBM (Orange)	0.955 [0.931, 0.972]	86.5 [82.9, 89.5]	100.0 [59.0, 100.0]	86.3 [82.7, 89.3]	0.463
SVM (MATLAB)	0.892 [0.859, 0.919]	78.2 [74.1, 81.9]	85.7 [42.1, 99.6]	78.1 [73.9, 81.8]	0.368
HbA1c	0.896 [0.865, 0.924]	68.5 [64.0, 72.8]	100.0 [59.0, 100.0]	68.0 [63.4, 72.4]	0.328
DM duration	0.765 [0.724, 0.804]	49.9 [45.1, 54.6]	100.0 [59.0, 100.0]	49.1 [44.3, 53.8]	0.007
External validation					
LR (ChatGPT-4)	0.835 [0.780, 0.881]	66.3 [59.8, 72.4]	100.0 [29.2, 100.0]	65.9 [59.3, 72.1]	Reference
RF (R)	0.851 [0.798, 0.895]	69.4 [63.0, 75.3]	100.0 [29.2, 100.0]	69.0 [62.5, 74.9]	0.414
GBM (Orange)	0.851 [0.798, 0.895]	71.6 [65.3, 77.3]	100.0 [29.2, 100.0]	71.2 [64.8, 77.0]	0.805
SVM (MATLAB)	0.757 [0.695, 0.811]	82.5 [76.9, 87.2]	66.7 [9.4, 99.1]	82.7 [77.2, 87.4]	0.308
HbA1c	0.821 [0.765, 0.869]	72.5 [66.2, 78.2]	100.0 [29.2, 100.0]	72.1 [65.8, 77.8]	0.819
DM duration	0.770 [0.710, 0.824]	60.3 [53.6, 66.6]	100.0 [29.2, 100.0]	59.7 [53.0, 66.2]	0.609

*CI* confidence interval, *DM* diabetes mellitus, *GBM* gradient boosting machine, *LR* logistic regression, *ROC-AUC* area under the receiver operating characteristic curve, *SVM* support vector machine

^*^Differences in ROC-AUC values compared to the logistic regression performed using ChatGPT-4

## Discussion

This study is the first to design an automated risk calculator, operational on a web browser, for predicting DR and DME risks using a LLM without the need for retinal imaging or a coding process. Access to ophthalmologists and retinal imaging equipment is still limited in most communities, making retinal examination difficult to perform in all health screening centers [22]. Our proposed method can efficiently address these difficulties in DR and DME screening. Our approach using ChatGPT-4 is highly cost-effective and can accelerate the clinical application of algorithms developed to screen DR and DME through a rapid development cycle and reflection of feedback.

Most clinicians have only encountered chronic disease risk analysis models in research papers, making it challenging to apply these analyses in clinical practice [23]. Clinicians need to enhance the efficiency and effectiveness of their clinical processes based on AI [7]. By following the methodology of this study, clinicians can use ChatGPT-4 to overcome barriers to coding skills, directly analyze risk factors, and develop prediction models. This allows for the creation of a risk calculator that can be conveniently used directly in clinical practice, enabling more patients to assess their disease risk and benefit more directly. In addition, ChatGPT-4 simplifies the process of updating calculators based on new data, allowing clinicians to create customized chronic disease risk calculators using data from their own institutions. The coding capabilities of LLM have already been confirmed and are actively used in many industry [24]. We expect that the development of automated calculators in the medical field will benefit many clinicians and patients with limited access to medical care.

Instead of focusing on retinal imaging, we proposed a new method that utilizes clinical and laboratory data to reveal patterns associated with complication of metabolic disease. By applying the numerical data-based calculator development approach for DR and DME proposed in this study, patients requiring retinal examinations can be effectively screened. Previous studies have identified demographic and biological risk factors for DR and DME through statistical analyses using national study data [25, 26]. Recently, machine learning approaches have been proposed to predict the occurrence of DR based on larger-scale data [4, 5]. Most studies have used complex tree-based algorithms like RF or XGBoost to improve performance [27, 28]. The calculator in this study is based on HTML and can operate as a web browser application, even offline, without a server connection. This means it can be used on any computer at a medical institution. Generally, developing and running a machine learning model requires initial development costs for GPU usage and ongoing server maintenance costs. However, an HTML-based calculator using ChatGPT-4 incurs no such development costs and is easy to maintain. Our experimental results showed that the prediction results of the regression analysis and machine learning were statistically similar. Therefore, clinicians will be able to calculate the risk of DR and DME in diabetic patients with accuracy and reliability using the calculator proposed in this study.

Several studies have consistently reported the difficulty of developing machine learning algorithms that show optimal performance across various external validation sets [29, 30]. In a disease prediction model, it is challenging to achieve both fitting and generalization of the data simultaneously [31]. Instead of large-scale validation of algorithms developed after collecting big data, another alternative could be for individual institutions to produce customized algorithms and apply them to clinical trials [18]. Using the method proposed in this study, individual organizations can easily create customized risk calculators with the data they collect. Each institution has a unique patient population and testing equipment. The prevalence and risk factors for diabetic complications may vary depending on socioeconomic environment, genetic background, and lifestyle. In this study, we used sample data from South Korea; however, it is possible to develop a customized risk calculator for DR and DME with higher performance by utilizing data collected from individual hospitals using ChatGPT-4. Previously, data analysis experts were required to develop individual algorithms [32]. However, our study showed that not only many data analysis processes but also the design of algorithms and creation of user interfaces could be replaced by ChatGPT-4. Researchers from other institutions and countries can use their data to develop customized risk calculators that can be easily used in clinical practice.

DR and DME are closely related and predictable based on systemic multi-factors such as hyperglycemia, hypertension, duration of diabetes, and genetic factor [25, 26, 33]. Increased neutrophil counts, reflecting a systemic inflammatory state, are also known to be associated with the development of DR [34]. The developed DR and DME risk calculators linearly combined known clinical risk factors and laboratory test results. Since there was no statistical difference in diagnostic performance between the nonlinear machine learning and linear logistic regression models, it appears that there is no specific nonlinear relationship between the variables in predicting DR and DME. In addition, the importance analysis from AI tools and the feature selection from ChatGPT-4’s regression analysis showed similar results.

Most studies using AI chatbots in the medical field have been limited to confirming diagnoses or plans using their knowledge [35, 36]. This study went beyond the scope of previous studies by demonstrating that direct statistical processing and software production were possible without coding. During our research with ChatGPT-4, we observed several advantages and disadvantages. The benefits of this method include ChatGPT-4’s ability to recognize data names and provide various insights. For example, it recognized the “glucose level” and “HbA1c” names, provided the normal range, and appropriately handled the imputation of missing data. In addition, the data summary and analysis processes were explained in the chat window. The feature selection and regression analysis processes were detailed, allowing the researcher to review for errors. A disadvantage of ChatGPT-4 was that the answers to the same questions are not always consistent. The algorithm of ChatGPT-4 uses internal randomness to generate various answers and select the optimal one [37]. In addition, hallucinations can occur if the prompt is nonspecific. When coding, unspecified items were often processed arbitrarily according to the context. While delegating the details of analysis and software to ChatGPT-4 was convenient, it sometimes produced hallucinations. Therefore, when developing a risk calculator using ChatGPT-4, it is essential to clearly specify the requirements for analysis methods and coding.

This study had several limitations. First, the cross-sectional nature of data collection in the KNHANES hinders the development of a prediction model for the future development of DR and DME [38]. Longitudinal follow-up data are required to build developmental prediction models; however, large-scale cross-sectional studies such as ours provide meaningful insights. Second, the data were collected from an East Asian country, raising uncertainty about the generalizability of our models to other countries or ethnic groups. It is recommended that ChatGPT-4 be used to establish individual calculation formulas with data specific to each ethnic group as both DR and DME risks may differ [39]. Third, our dataset includes variables such as blood glucose levels and BMI measurements, which fluctuated during data collection. These variations can introduce noise into the predictions and reduce accuracy.

Another notable limitation is that the dataset included only diabetic patients, which restricts the applicability of the risk calculator to broader populations containing both diabetic and non-diabetic individuals. In mixed populations, the overall accuracy of predicting diabetic retinopathy is expected to increase, but sensitivity and specificity may be significantly affected by the presence of other ophthalmic conditions with overlapping features, such as hypertensive retinopathy. To address these challenges, future studies should test the calculator on datasets representing more diverse populations. This would help evaluate its performance, account for the impact of coexisting conditions, and refine the algorithm for wider clinical use.

## Conclusion

The risk calculator developed using ChatGPT-4 demonstrated moderate accuracy in predicting DR and DME, with performance metrics comparable to traditional machine learning algorithms. Additionally, this calculator runs in a web browser using ChatGPT-4, allowing clinicians to quickly identify patients who require detailed retinal examinations at the point of care. Our approach offers an easily accessible tool that enables risk prediction of DM and DME in routine health check-up screening for patients with diabetes. However, it is important to acknowledge that the dataset used comprised only diabetic patients, which may limit the generalizability of the model to broader populations. Additionally, the reliance on self-reported medical history and laboratory data, which can be subject to variability, may affect the precision of predictions. Future research should focus on validating this approach in diverse populations and exploring the integration of more comprehensive clinical data to enhance predictive performance.

## Supplementary Information

Supplementary material 1: Material 1. HTML codes generated by ChatGPT-4. Material 2. SHAP feature importances from random forest models developed using R. Material 3. Feature importance from gradient boosting machine models developed using Orange Data Mining.

## Data Availability

The dataset is publicly available for research at https://knhanes.kdca.go.kr/knhanes/eng/index.do.

## Abbreviations

ALT: Alanine aminotransferase
AST: Aspartate aminotransferase
BMI: Body mass index
BUN: Blood urea nitrogen
CSME: Clinically significant macular edema
DBP: Diastolic blood pressure
DME: Diabetic macular edema
DR: Diabetic retinopathy
GBM: Gradient boosting machine
HbA1c: Glycated hemoglobin
HTML: Hypertext Markup Language
KNHANES: Korea National Health and Nutrition Examination Survey
LLM: Large language model
OCT: Optical coherence tomography
RF: Random forest
ROC-AUC: Receiver operating characteristic area under the curve
SBP: Systolic blood pressure
SVM: Support vector machine
WBC: White blood cell count
