# Supplementary material for: Automated and code-free development of a risk calculator using ChatGPT-4 for predicting diabetic retinopathy and macular edema without retinal imaging
Source: Int J Retina Vitreous. 2025 Jan 31;11:11. doi: 10.1186/s40942-025-00638-9 (PMC11786427; doi:10.1186/s40942-025-00638-9)
Supplement: Supplementary file 1 — Supplementary material 1: Material 1. HTML codes generated by ChatGPT-4. Material 2. SHAP feature importances from random forest models developed using R. Material 3. Feature importance from gradient boosting machine models developed using Orange Data Mining. [file 40942_2025_638_MOESM1_ESM.pdf]

Supplementary Materials

Supplementary Material 1. HTML codes generated by ChatGPT-4

```
<!DOCTYPE html>
<html lang="en">
<head>
    <meta charset="UTF-8">
    <meta name="viewport" content="width=device-width, initial-scale=1.0">
    <title>Diabetic Retinopathy (DR) and Diabetic Macular Edema (DME) Risk Calculator</title>
    <style>
        body {
            font-family: Arial, sans-serif;
            display: flex;
            justify-content: center;
            align-items: center;
            height: 100vh;
            margin: 0;
            background-color: #f0f0f0;
        }
        .calculator-container {
            width: 800px;
            height: 800px;
            background: white;
            border-radius: 10px;
            box-shadow: 0 0 10px rgba(0, 0, 0, 0.1);
            padding: 20px;
            box-sizing: border-box;
            display: flex;
            flex-direction: column;
            justify-content: space-between;
        }
        .title-frame, .input-frame, .output-frame {
            border: 1px solid #ccc;
            border-radius: 10px;
            padding: 15px;
            background: #fff;
        }
        .title-frame {
            text-align: center;
            font-size: 1.2em;
            font-weight: bold;
        }
        .input-frame {
            font-size: 1em;
            flex: 1;
            display: flex;
```

```css
        flex-direction: column;
        justify-content: center;
    }
    .output-frame {
        font-size: 1em;
    }
    .frame-title {
        font-size: 1.1em;
        font-weight: bold;
        margin-bottom: 10px;
    }
    .input-group {
        margin-bottom: 15px;
    }
    .input-group label {
        display: block;
        margin-bottom: 5px;
    }
    .input-group input {
        width: calc(100% - 20px);
        padding: 10px;
        border-radius: 5px;
        border: 1px solid #ccc;
        box-sizing: border-box;
    }
    .input-group input[type="number"] {
        -moz-appearance: textfield;
    }
    .input-group input[type="number"]::-webkit-outer-spin-button,
    .input-group input[type="number"]::-webkit-inner-spin-button {
        -webkit-appearance: none;
        margin: 0;
    }
    .input-columns {
        display: flex;
        justify-content: space-between;
    }
    .input-column {
        width: 30%;
    }
    .button-group {
        text-align: center;
    }
    .button-group button {
        padding: 10px 20px;
        border: none;
        border-radius: 5px;
        background-color: #007bff;
```

```
            color: white;
            font-size: 1em;
            cursor: pointer;
        }
        .button-group button:hover {
            background-color: #0056b3;
        }
    </style>
</head>
<body>
    <div class="calculator-container">
        <div class="title-frame">
            Diabetic Retinopathy (DR) and Diabetic Macular Edema (DME) Risk Calculator
        </div>
        <div class="input-frame">
            <div class="frame-title">Medical History and Laboratory Data</div>
            <div class="input-columns">
                <div class="input-column">
                    <div class="input-group">
                        <label for="age">Age (years)</label>
                        <input type="number" id="age" required>
                    </div>
                    <div class="input-group">
                        <label for="DM_insulin">DM Insulin (0: No, 1: Yes)</label>
                        <input type="number" id="DM_insulin" min="0" max="1" required>
                    </div>
                    <div class="input-group">
                        <label for="DM_duration">DM Duration (years)</label>
                        <input type="number" id="DM_duration" required>
                    </div>
                    <div class="input-group">
                        <label for="DM_po_med">DM PO Med (0: No, 1: Yes)</label>
                        <input type="number" id="DM_po_med" min="0" max="1" required>
                    </div>
                </div>
                <div class="input-column">
                    <div class="input-group">
                        <label for="HE_sbp">HE SBP (mmHg)</label>
                        <input type="number" id="HE_sbp" required>
                    </div>
                    <div class="input-group">
                        <label for="HE_BMI">HE BMI (kg/m²)</label>
                        <input type="number" id="HE_BMI" step="0.1" required>
                    </div>
                    <div class="input-group">
                        <label for="HE_glu">HE Glu (mg/dL)</label>
                        <input type="number" id="HE_glu" required>
                    </div>
```

```
                    <div class="input-group">
                        <label for="HE_HbA1c">HE HbA1c (%)</label>
                        <input type="number" id="HE_HbA1c" step="0.1" required>
                    </div>
                </div>
                <div class="input-column">
                    <div class="input-group">
                        <label for="HE_HB">HE HB (g/dL)</label>
                        <input type="number" id="HE_HB" step="0.1" required>
                    </div>
                    <div class="input-group">
                        <label for="HE_crea">HE Crea (mg/dL)</label>
                        <input type="number" id="HE_crea" step="0.01" required>
                    </div>
                    <div class="input-group">
                        <label for="HE_WBC">HE WBC (×10³/μL)</label>
                        <input type="number" id="HE_WBC" step="0.01" required>
                    </div>
                    <div class="input-group">
                        <label for="HE_Bplt">HE Bplt (×10³/μL)</label>
                        <input type="number" id="HE_Bplt" required>
                    </div>
                </div>
            </div>
            <div class="button-group">
                <button onclick="calculateRisk()">Calculate Risk</button>
            </div>
        </div>
        <div class="output-frame">
            <div class="frame-title">DR and DME Risk Results</div>
            <div id="results"></div>
        </div>
    </div>

    <script>
        function calculateRisk() {
            // Retrieve input values
            const age = parseFloat(document.getElementById('age').value);
            const DM_insulin = parseFloat(document.getElementById('DM_insulin').value);
            const DM_duration = parseFloat(document.getElementById('DM_duration').value);
            const DM_po_med = parseFloat(document.getElementById('DM_po_med').value);
            const HE_sbp = parseFloat(document.getElementById('HE_sbp').value);
            const HE_BMI = parseFloat(document.getElementById('HE_BMI').value);
            const HE_glu = parseFloat(document.getElementById('HE_glu').value);
            const HE_HbA1c = parseFloat(document.getElementById('HE_HbA1c').value);
            const HE_HB = parseFloat(document.getElementById('HE_HB').value);
            const HE_crea = parseFloat(document.getElementById('HE_crea').value);
            const HE_WBC = parseFloat(document.getElementById('HE_WBC').value);
```

```
            const HE_Bplt = parseFloat(document.getElementById('HE_Bplt').value);

            // Calculate E_DR risk score
            const logit_E_DR = -3.1697 - 0.0201 * age + 1.0679 * DM_insulin + 0.0385 * DM_duration + 0.6820 * DM_po_med + 0.0150 * HE_sbp - 0.0433 * HE_BMI + 0.0065 * HE_glu + 0.2048 * HE_HbA1c - 0.0738 * HE_HB;
            const P_E_DR = 1 / (1 + Math.exp(-logit_E_DR));
            const DR_risk_score = (P_E_DR * 100).toFixed(2);
            const DR_risk_group = P_E_DR >= 0.212 ? 'High-risk' : 'Low-risk';

            // Calculate E_DME risk score
            const logit_E_DME = -7.7312 + 0.0577 * DM_duration + 0.0236 * HE_sbp + 0.5337 * HE_HbA1c - 0.3655 * HE_HB + 0.7647 * HE_crea + 0.2003 * HE_WBC - 0.0055 * HE_Bplt;
            const P_E_DME = 1 / (1 + Math.exp(-logit_E_DME));
            const DME_risk_score = (P_E_DME * 100).toFixed(2);
            const DME_risk_group = P_E_DME >= 0.017 ? 'High-risk' : 'Low-risk';

            // Display results
            document.getElementById('results').innerHTML = `
                <p>DR Risk Score: ${DR_risk_score}%</p>
                <p>DR Risk Group: ${DR_risk_group}</p>
                <p>DME Risk Score: ${DME_risk_score}%</p>
                <p>DME Risk Group: ${DME_risk_group}</p>
            `;
        }
    </script>
</body>
</html>
```

Supplementary Material 2. SHAP feature importances from random forest models developed using R.

(A) Feature importance for DR prediction

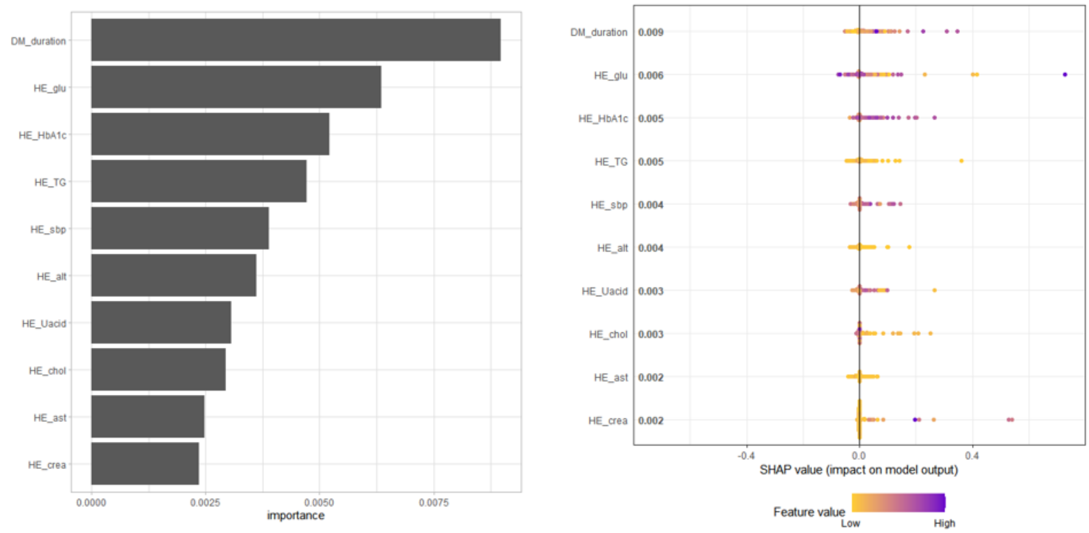

(B) Feature importance for DME prediction

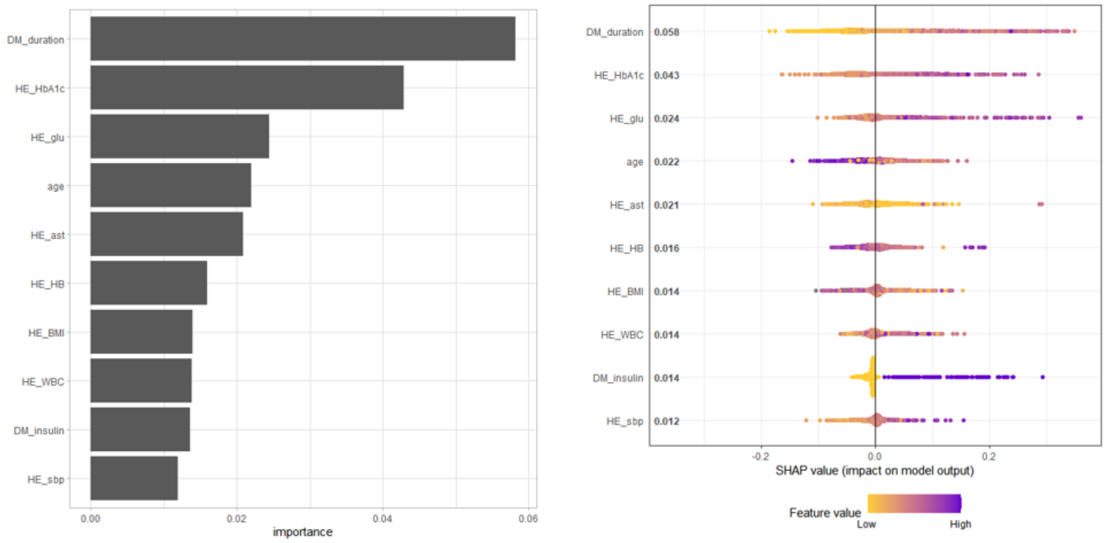

Supplementary Material 3. Feature importance from gradient boosting machine models developed using Orange Data Mining.

(A) Orange Data Mining

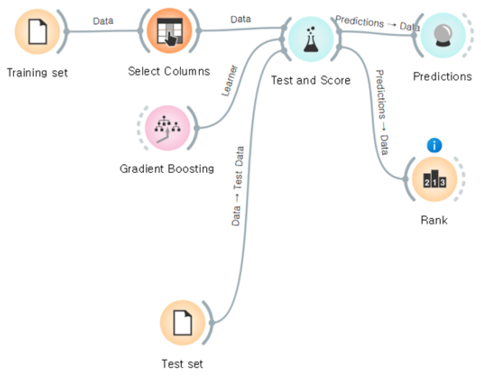

(B) Feature importance for DR prediction

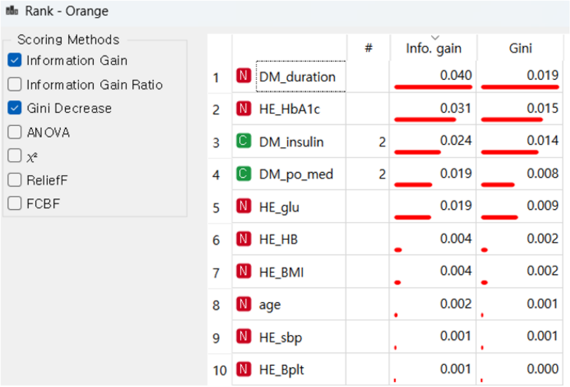

Rank - Orange

Scoring Methods

- [x] Information Gain
- [ ] Information Gain Ratio
- [x] Gini Decrease
- [ ] ANOVA
- [ ] χ^2
- [ ] ReliefF
- [ ] FCBF

			#	Info. gain	Gini
1	N	DM_duration		0.040	0.019
2	N	HE_HbA1c		0.031	0.015
3	C	DM_insulin	2	0.024	0.014
4	C	DM_po_med	2	0.019	0.008
5	N	HE_glu		0.019	0.009
6	N	HE_HB		0.004	0.002
7	N	HE_BMI		0.004	0.002
8	N	age		0.002	0.001
9	N	HE_sbp		0.001	0.001
10	N	HE_Bplt		0.001	0.000

(C) Feature importance for DME prediction

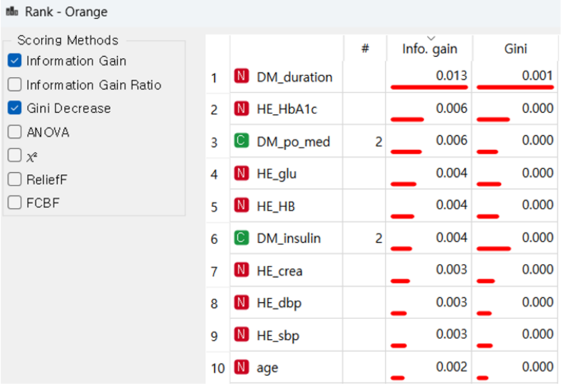

Rank - Orange

Scoring Methods

- [x] Information Gain
- [ ] Information Gain Ratio
- [x] Gini Decrease
- [ ] ANOVA
- [ ] χ^2
- [ ] ReliefF
- [ ] FCBF

			#	Info. gain	Gini
1	N	DM_duration		0.013	0.001
2	N	HE_HbA1c		0.006	0.000
3	C	DM_po_med	2	0.006	0.000
4	N	HE_glu		0.004	0.000
5	N	HE_HB		0.004	0.000
6	C	DM_insulin	2	0.004	0.000
7	N	HE_crea		0.003	0.000
8	N	HE_dbp		0.003	0.000
9	N	HE_sbp		0.003	0.000
10	N	age		0.002	0.000
